# Andrographolide Inhibits Lytic Reactivation of Epstein-Barr Virus by Modulating Transcription Factors in Gastric Cancer

**DOI:** 10.3390/microorganisms9122561

**Published:** 2021-12-10

**Authors:** Praphatson Malat, Tipaya Ekalaksananan, Chukkris Heawchaiyaphum, Supawadee Suebsasana, Sittiruk Roytrakul, Yodying Yingchutrakul, Chamsai Pientong

**Affiliations:** 1 Department of Microbiology, Faculty of Medicine, Khon Kaen University, Khon Kaen 40002, Thailand; phatson88@gmail.com (P.M.); tipeka@kku.ac.th (T.E.); jukkris.003@gmail.com (C.H.); 2 HPV & EBV and Carcinogenesis Research Group, Khon Kaen University, Khon Kaen 40002, Thailand; 3 Department of Pharmaceutical Sciences, Faculty of Pharmacy, Thammasat University, Bangkok 10200, Thailand; hnungnet@yahoo.com; 4 Genome Technology Research Unit, Proteomics Research Laboratory, National Center for Genetic Engineering and Biotechnology (BIOTEC), Pathum Thani 12120, Thailand; sittiluk@biotec.or.th (S.R.); yodying.yin@nstda.or.th (Y.Y.)

**Keywords:** andrographolide, EBV, EBV lytic reactivation, transcriptional factor, MEF2D, SP1, histone modifications, HDAC6

## Abstract

Andrographolide is the principal bioactive chemical constituent of *Andrographis paniculata* and exhibits activity against several viruses, including Epstein–Barr virus (EBV). However, the particular mechanism by which andrographolide exerts an anti-EBV effect in EBV-associated gastric cancer (EBVaGC) cells remains unclear. We investigated the molecular mechanism by which andrographolide inhibits lytic reactivation of EBV in EBVaGC cells (AGS-EBV cell line) using proteomics and bioinformatics approaches. An andrographolide treatment altered EBV protein-expression patterns in AGS-EBV cells by suppressing the expression of EBV lytic protein. Interestingly cellular transcription factors (TFs), activators for EBV lytic reactivation, such as MEF2D and SP1, were significantly abolished in AGS-EBV cells treated with andrographolide and sodium butyrate (NaB) compared with NaB-treated cells. In contrast, the suppressors of EBV lytic reactivation, such as EZH2 and HDAC6, were significantly up-regulated in cells treated with both andrographolide and NaB compared with NaB treatment alone. In addition, bioinformatics predicted that HDAC6 could interact directly with MEF2D and SP1. Furthermore, andrographolide significantly induced cell cytotoxicity and apoptosis of AGS-EBV cells by induction of apoptosis-related protein expression. Our results suggest that andrographolide inhibits EBV lytic reactivation by inhibition of host TFs, partially through the interaction of HDAC6 with TFs, and induces apoptosis of EBVaGC cells.

## 1. Introduction

EBV is a ubiquitous gamma-herpesvirus and is strongly associated with the development of several human cancers, including Burkitt’s lymphoma (BL), Hodgkin’s lymphoma, nasopharyngeal carcinoma (NPC), and EBVaGC. EBVaGC is a subtype of gastric adenocarcinoma (GC) and accounts for approximately 10% of all GCs. EBV establishes two alternative modes of infection in the host cell, latent and lytic infection. Latent infection plays an essential role in carcinogenesis through the activities of latent-stage proteins, especially LMP1. Upon reactivation induced by various stimuli, EBV undergoes three consecutive lytic stages, the immediate early (IE), early (E), and late (L) stages. The IE proteins, Zta and Rta, activate transcription from the promoters of E genes, which trigger EBV genomic DNA replication from the origin of replication. Then the L genes that encode structural proteins are expressed, followed by viral genome packaging into infectious virion particles. In recent years, the EBV lytic cycle has been reported to contribute to carcinogenesis of several cancer types [1].

The regulation of EBV lytic reactivation is initially determined by the availability of viral proteins and host TFs that can either suppress or activate the *BZLF1* promoter (Zp) and BRLF1 promoter (Rp) [2,3]. Zp is activated by various TFs, including myocyte enhancer factor 2 (MEF2), specificity protein 1 (SP1), b-Zip type transcription factors, and also histone acetylation and histone 3 lysine 4 trimethyl (H3K4me3). In contrast, suppression of the Zp is mediated by transcriptional suppressors, such as ZEB, ZIIR-BP, and jun dimerization protein 2 (JDP2), and the histone modifications H3K27me3, H3K9me2/3, and H4K20me3 [3].

Andrographolide exhibits various medicinal properties, such as anti-inflammatory, antioxidant, anti-bacterial, anti-retroviral, anti-tumor, anti-diabetic, anti-malarial, anti-hypertension, anti-rheumatism, hepatoprotective, immunostimulant, anti-cancer, anti-angiogenic, and anti-viral activities [4,5,6]. Andrographolide extracted from *Andrographis paniculata* (Burm. f.) Nees (family *Acanthaceae*) contains diverse compounds such as labdane diterpenoid lactones, flavonoids, and miscellaneous compounds and is widely used as a traditional medicine in Asian countries [5,7]. Anti-viral activity of andrographolide has been shown for a number of viruses including herpes simplex virus [8], dengue virus (DENV) [9], chikungunya virus (CHIKV) [10], human papillomavirus (HPV) [11], hepatitis B virus (HBV) [12], and influenza A [13].

Andrographolide can inhibit EBV lytic reactivation by suppressing EBV lytic genes and the activity of Zp [14]. However, the mechanism by which andrographolide exerts its antiviral effect remains unclear. Therefore, we aimed to explore this question in gastric cancer cell lines by using proteomics and bioinformatics approaches. Here, we demonstrated that andrographolide inhibits EBV lytic reactivation through the direct interaction of HDAC6 with host TFs. In addition, andrographolide also induced apoptosis of EBVaGC cells.

## 2. Materials and Methods

### 2.1. Cells Lines, Compound, and Experimental Design

**Figure 1:**
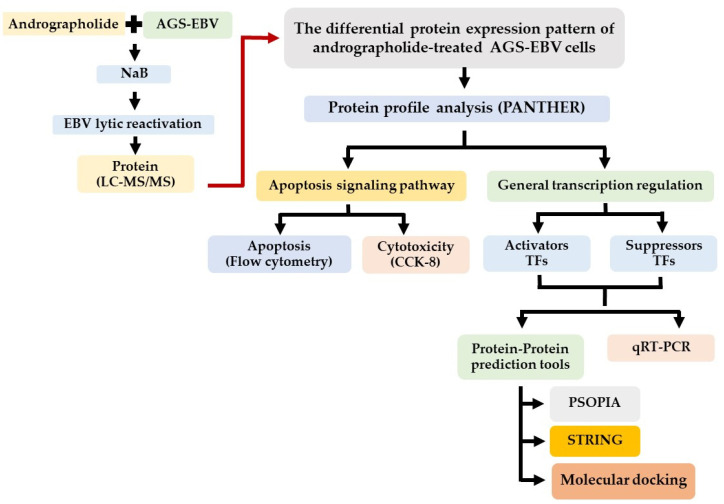
Schematic representation of experimental design.

The GC cell lines, AGS and AGS-EBV (kindly provided by Prof. Hironori Yoshiyama, Shimane University, Japan) were cultured in RPMI 1640 medium containing 10% FBS and antibiotics (streptomycin and penicillin). The andrographolide compound was prepared as previously described [15] by the Department of Pharmaceutical Sciences, Faculty of Pharmacy, Thammasat University, Bangkok, Thailand. The experimental design is shown in Figure 1.

### 2.2. Investigation of the Induction of Cell Cytotoxicity by Andrographolide

To investigate whether andrographolide induces cytotoxicity in GC cell lines. AGS and AGS-EBV cells (1 × 10^5^ cells/mL) were seeded into 96-well plates and pre-incubated at 37 °C, 5% CO_2_ for 24 h. After incubation, culture medium was removed, and cells were subjected to one of three treatments: andrographolide alone, NaB alone, or a combination of andrographolide and NaB. Cells were then incubated at 37 °C, 5% CO_2_ for 24 and 48 h. Cell viability was determined using the CCK-8 assay [16].

### 2.3. Andrographolide Treatment and Protein Extraction by TRIzol Reagent

The EBV-infected cell line was treated with andrographolide at 25% cytotoxic contraction (CC_25_) or concurrently treated with NaB and andrographolide at CC_25_. A control group did not receive either treatment. Total protein was extracted using TRIzol™ reagent (Invitrogen, Waltham, MA, USA) according to the manufacturer’s instructions. The total protein concentration was determined using Bio-Rad protein assay-dye reagent concentration kit. Protein samples were analyzed on 12.5% sodium dodecyl sulfate–polyacrylamide gel electrophoresis (SDS–PAGE).

### 2.4. Proteomic Analysis by Mass Spectrometry

Protein content was determined using the Lowry method [17] and bovine serum albumin (BSA) as standard. Total protein (10 μg) was packed with 12.5% poly acrylamide, and in-gel digestion was performed as described previously [18].

The tryptic peptide samples were prepared for injection into an Ultimate 3000 Nano/Capillary LC System (Thermo Scientific, Winsford, UK) coupled to a Hybrid quadrupole Q-Tof impact II™ (Bruker Daltonics, Billerica, MA, USA) equipped with a nano-captive spray ion source. Briefly, peptides were enriched on a µ-precolumn 300 µm i.d. × 5 mm C18 Pep Map 100, 5 µm, 100 A (Thermo Scientific, Winsford, UK), separated on a 75 μm I.D. × 15 cm, and packed with Acclaim Pep Map RSLC C18, 2 μm, 100 A, nanoViper (Thermo Scientific, Winsford, UK). Solvent A and B containing 0.1% formic acid in water and 0.1% formic acid in 80% acetonitrile, respectively, were applied on the analytical column. A gradient of 5–55% of solvent B was used to elute the peptides at a constant flow rate of 0.30 μL/min for 30 min. Electrospray ionization was carried out at 1.6 kV using the Captive Spray. Mass spectra (MS) and MS/MS spectra were obtained in the positive-ion mode over the range (*m*/*z*) 150–2200 (Compass 1.9 software, Bruker Daltonics).

MaxQuant 1.6.1.12 was used to quantify the proteins in individual samples using the Andromeda search engine to correlate MS/MS spectra to the Uniprot database. The following parameters were used for data processing: maximum of two missed cleavages, mass tolerance of 20 ppm for main search, trypsin as digesting enzyme, carbamidomethylating of cysteine as fixed modification, and the oxidation of methionine and acetylation of the protein N-terminus as variable modifications. Only peptides with a minimum of seven amino acids, as well as at least one unique peptide, were required for protein identification. Only proteins with at least two peptides, and at least one unique peptide, were considered as being identified and used for further data analysis.

### 2.5. Data Analysis and Molecular Docking

The functional annotation of the unique proteins was performed by PANTHER [19]. The differentially expressed proteins among the conditions of cells were analyzed by a jvenn web tool (http://jvenn.toulouse.inra.fr, accessed on 5 January 2021) [20]. The PPI network was constructed by STRING (https://string-db.org/, accessed on 24 August 2021) [21]. The PPI networks were visualized using Cytoscape software (version 3.8.2).

To further confirm protein–protein interaction, molecular docking was performed. The sequences of candidate proteins were submitted to SWISS-MODEL [22], protein prediction software, to build up predicted 3D structures. The stereochemistry of proteins was evaluated by Ramachandran Plot Analysis (https://zlab.umassmed.edu/bu/rama/, accessed on 29 November 2021). All ionizable amino acid residues in predicted models were protonated at pH 7.4 by using H++ 1.0 (http://biophysics.cs.vt.edu/, accessed on 29 November 2021). Molecular docking simulation was performed by the web-based service ClusPro [23] to identify the interaction of the candidate proteins. The PyMOL was used to perform visualization of 3D models. The coefficient weights of protein binding was calculated from E = 0.40Erep + (−0.40)Eatt + 600Eelec + 1.00EDARS [24].

### 2.6. Quantification of Gene Expression by qRT-PCR

Total RNA was extracted from cells that were treated for 24 h with either NaB or andrographolide or a combination of both using TRIzol™ reagent (Invitrogen, Waltham, MA, USA) according to the manufacturer’s instructions. The expression of host TF genes (*MEF2D* and *SP1*) and *HDACs* (*HDAC6* and *HDAC9*) was determined by qRT-PCR using SsoAdvancedTM SYBR^®^ Green Supermix (Bio-Rad, Hercules, CA, USA) in the QuantStudio 6 Flex Real-Time PCR System (Applied Biosystems, Foster City, CA, USA). The *GAPDH* gene was detected as an internal control. The primer sequences used in the present study are listed in Table 1.

### 2.7. Quantification of Cell Apoptosis by Flow Cytometry

The effect of andrographolide on cell apoptosis was analyzed using flow cytometry. Cells of the AGS-EBV and AGS cell lines (2.5 × 10^5^ cells/well) were seeded into a 6-well plate and incubated at 37 °C, 5% CO_2_ for 18 h to allow the cells to adhere to the plate. Cells were then either treated with NaB or andrographolide or a combination of both. Cells were then incubated at 37 °C, 5% CO_2_ for 18 h. After incubation, cells were collected by trypsinization and centrifugation. Apoptotic cells were detected using Dead Cell Apoptosis Kits (Invitrogen, Carlsbad, CA, USA) using flow cytometry (FACS Canto II; BD bioscience, Franklin Lakes, NJ, USA), and data were processed using FlowJo software (Flowjo, Treestar Inc., Ashland, OR, USA). Cells treated with 50 nM of staurosporine and 0.1% DMSO were used as positive controls and negative controls, respectively.

### 2.8. Statistical Analysis

All data were analyzed using the GraphPad Prism program (GrapPad Software Inc., San Diego, CA, USA). Real-time PCR data was evaluated using independent sample t-tests. Differences were considered statistically significant at a *p*-value of less than 0.05.

## 3. Results

### 3.1. Andrographolide Alters Protein Expression Profile of EBVaGC Cells

**Figure 2:**
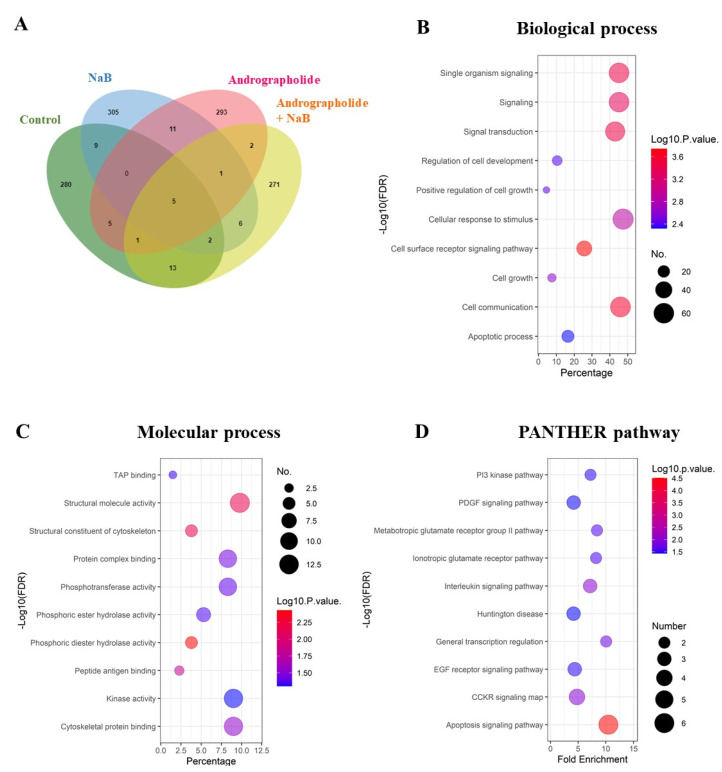
Andrographolide treatment modulates host gene expression in EBV-infected cells and functional annotation of proteins in cells with EBV lytic activation condition. (**A**) The unique proteins were visualized using a Venn diagram. (**B**) The functional annotation was determined in two aspects: biological processes; (**C**) molecular function by PANTHER. (**D**) The biological pathways were involved with up-regulated proteins.

To confirm whether andrographolide suppresses EBV lytic reactivation, AGS-EBV cells were pre-treated with andrographolide at CC_25_, 34.40 μM for 3 h, and subsequently incubated with or without NaB for 48 h. Total proteins were extracted and subjected to LC–MS/MS. As shown in Figure 2A, the Venn diagram analysis showed proteins unique to a single treatment and overlapping proteins in each condition. Because we wished to examine the effect of andrographolide on the inhibition of EBV lytic reactivation, we analyzed further the proteins unique to cells treated with andrographolide and NaB.

The functional annotation of proteins that were only expressed in cells subjected to concurrent treatment of andrographolide and NaB was done using PANTHER. These differentially expressed proteins were involved in biological processes, such as cell-surface receptor signaling pathway, cell communication, signaling transduction, and others (Figure 2B). In terms of molecular function, the proteins were involved in phosphoric diester hydrolase activity, structural molecular activity, structural constituents of the cytoskeleton, and so on (Figure 2C). In addition, these unique proteins were significantly enriched in the apoptosis signaling pathway, PI3 kinase pathway, PDGF signaling pathway, and other pathways (Figure 2D).

### 3.2. Andrographolide Inhibits EBV Lytic Reactivation by Inhibition of TFs Expression

**Figure 3:**
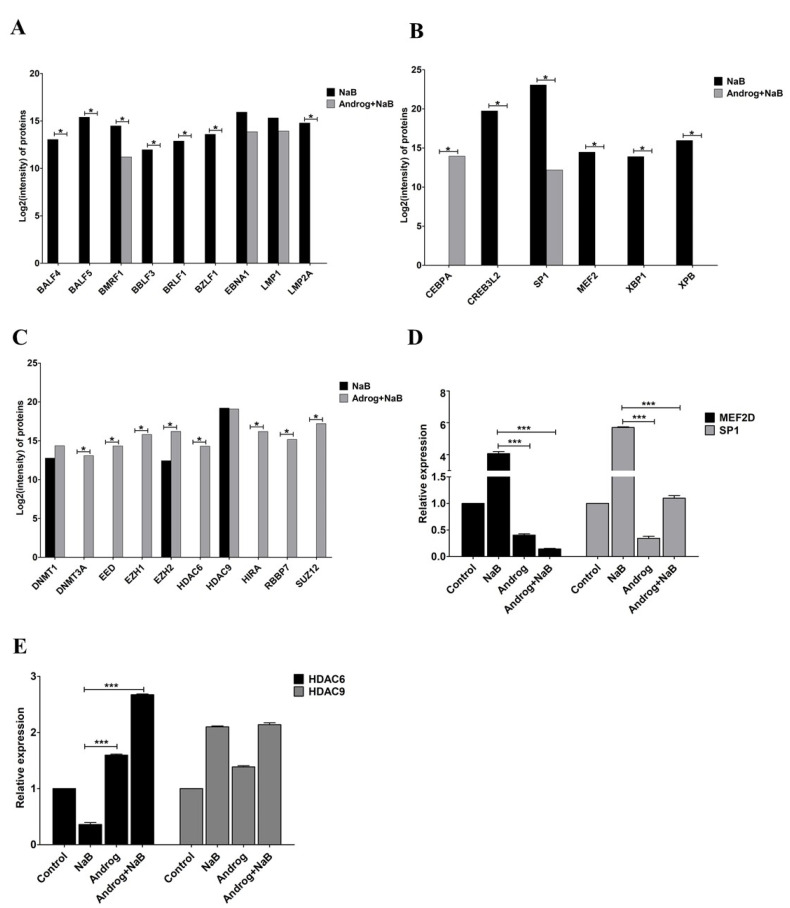
Andrographolide inhibits the expression of EBV lytic protein and host TFs. Cells were either treated with NaB or a combination of andrographolide and NaB, and the expression of EBV lytic genes and host TFs was examined using LC–MS/MS. (**A**) Lytic and latent genes; (**B**) activators; (**C**) suppressors and qRT-PCR; (**D**) *MEF2D* and *SP1*; (**E**) *HDAC6* and *HDAC9*. *: *p* < 0.05 and ***: *p* < 0.001.

Previously, we found that andrographolide could inhibit EBV lytic reactivation by inhibition of EBV lytic protein expression and of viral particle production. In the present study, we investigated this further and found that expression of EBV lytic proteins, including BALF4, BALF5, BMRF1, BBLF3, BRLF1 and BZLF1 was significantly inhibited by andrographolide (Figure 3A) In addition, the expression of LMP2A was completely inhibited in cells treated with andrographolide and NaB, However, the expression of EBNA1 and LMP1, remained unchanged. This proteomics result also suggests that andrographolide inhibits EBV lytic reactivation by inhibition of lytic protein expression.

Emerging evidence demonstrates that various host TFs could regulate lytic reactivation of EBV. Therefore, we examined the expression of host TFs using LC–MS/MS. We found that the expression of CREB3L2, MEF2D, SP1, XBP1 and XPB was decreased in AGS-EBV cells treated with the combination of andrographolide and NaB compared with NaB treatment alone. Whereas the expression of CEBPA was increased in AGS-EBV cells treated with the combination of andrographolide and NaB compared with NaB treatment alone (Figure 3B). In addition, we also examined the expression of suppressors for EBV lytic reactivation. The expression of protein-associated with epigenetic mechanisms, DNA methylation (as represented by DNMT3A), histone acetylation (HDAC6) and histone methylation (EED, EZH1, EZH2, RBBP7, SUZ12 and HIRA), was significantly increased in AGS-EBV cells treated with the combination of andrographolide and NaB when compared with NaB treatment alone. Whereas DNMT1 was slightly increased in AGS-EBV cells treated with a combination of andrographolide and NaB when compared with NaB treatment alone. The expression of HDAC9 in AGS-EBV cells treated with a combination of andrographolide and NaB was comparable to NaB treatment alone (Figure 3C).

The expression of activators (*MEF2D* and *SP1*) and suppressors (*HDAC6* and *HDAC9*) of EBV lytic replication was determined by qRT-PCR. Similar to protein levels, the expression of *MEF2D* and *SP1* was significantly decreased in AGS-EBV cells treated with andrographolide and NaB compared with NB-treated AGS-EBV cells (Figure 2D). On the other hand, the expression of *HDAC6* was dramatically increased in AGS-EBV cells treated with andrographolide and NaB compared with NaB-treated cells. Consistent with mRNA level, the expression of *HDAC9* was not different in AGS-EBV cells treated with andrographolide and NaB compared with NaB-treated cells (Figure 3E). These results suggest that andrographolide inhibits EBV lytic reactivation by inhibition of TF expression, possibly, through epigenetic mechanisms, especially histone modification.

### 3.3. Andrographolide Modulates TFs Expression through the Direct Interaction of HDAC6 with TFs

**Figure 4:**
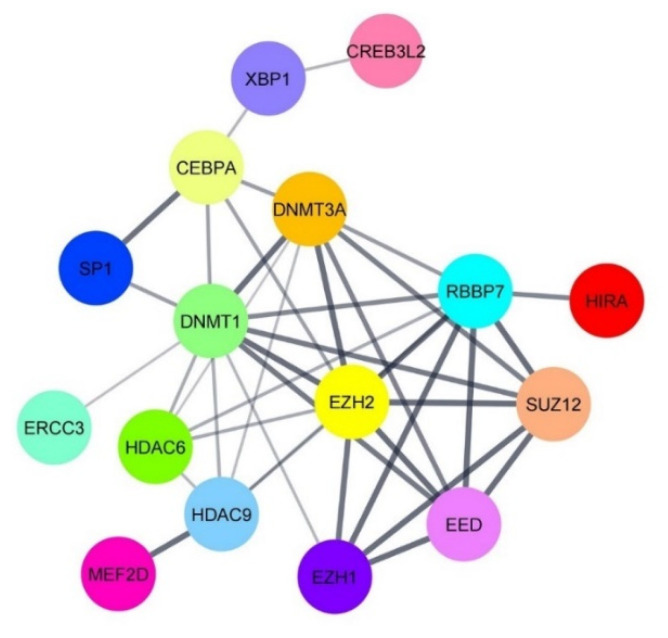
The PPI network of activators and suppressors of EBV lytic reactivation. The PPI network was constructed by STRING and visualized using Cystoscope software.

It is well documented that *HDACs* epigenetically modulate the expression of target genes [25,26]. Host TFs can either directly target DNA or undergo various post-translational modifications to alter gene expression. In this manner, *HDACs* negatively modulate transcription through forming a complex with TFs or by directly regulating the transcription of TFs [27]. In the present study, we found that *HDAC6* was significantly up-regulated in AGS-EBV cells treated with the combination of andrographolide and NaB. On the contrary, the expression of host TFs regulating EBV lytic reactivation, *MEF2D* and *SP1* was decreased in the combination of andrographolide and NaB treatment. Therefore, we hypothesized that HDACs can interact directly with such TFs. To test our hypothesis, we predicted the protein–protein interaction (PPI) using the PPI prediction tool, PSOPIA, and found that the probabilities that HDAC6 directly interacts with MEF2D and SP1 were 99.22% and 34.17%, respectively. In case of HDAC9, the probabilities were 99.91% and 90.11%, respectively (Table 2). In agreement with this, the PPI network constructed by STRING also showed that activators and suppressors of EBV lytic reactivation could interact with each other (Figure 4).

**Figure 5:**
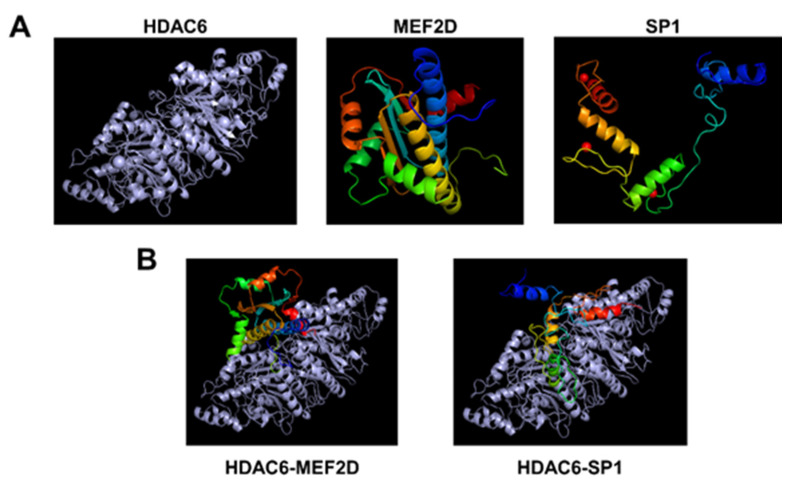
The molecular modeling and docking analysis for HDAC6, MEF2D, and SP1. (**A**) The 3D structure alignment of HDAC6, MEF2D, and SP1. (**B**) Molecular docking of HDAC6 with MEF2D and SP1. The gray color shows HDAC6 protein, and the rainbow shows MEF2 and SP1 proteins.

To further confirm whether HDAC6 can directly interact with MEF2D and SP1, molecular docking simulation was performed. As expected, molecular modeling and docking analysis suggests that HDAC6 could interact with the MEF2D and SP1 via electrostatic interactions and hydrogen bond formations (Figure 5, Table S1). The coefficient weights of the lowest energy of each interaction were −929.1 and −1104.1, respectively. Thus, these results indicate that HDAC6 interacts directly with MEF2D and SP1 and may disrupt the transcriptional activity of MEF2D and SP1 to activate the EBV lytic reactivation.

### 3.4. Andrographolide Induces Cell Cytotoxicity in an EBV-Associated Gastric Cancer Cell Line

**Figure 6:**
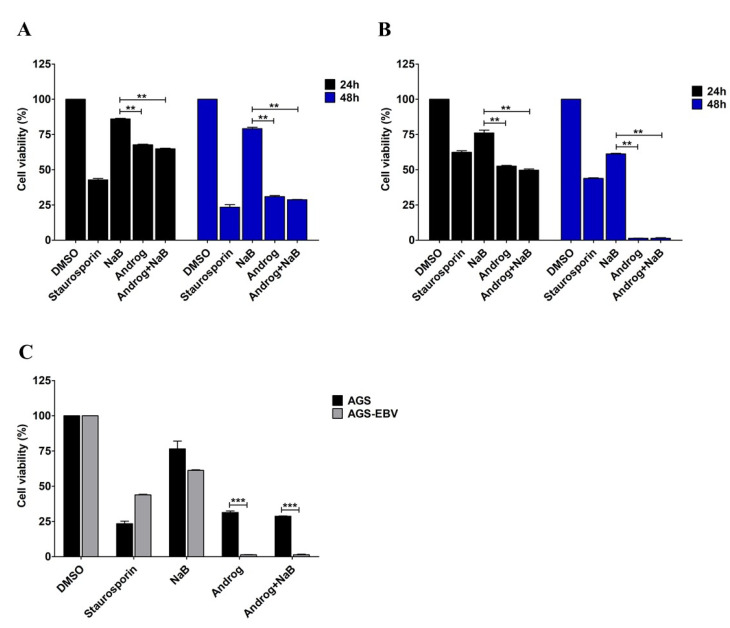
Andrographolide induces cytotoxicity in the EBVaGC cell line. AGS and AGS-EBV cells were either treated with NaB or andrographolide or a combination of both for 24 and 48 h. Viability of AGS cells (**A**) and of AGS-EBV cells (**B**) was evaluated using the CCK-8 assay. (**C**) The comparison of viability of AGS and AGS-EBV cells after treatment with NaB or andrographolide or the combination of both for 48 h. Staurosporin was used as a positive control for cell cytotoxicity. **: *p* < 0.01 and ***: *p* < 0.001.

Previously, we determined cytotoxic concentrations (CC_50_, CC_25_, and CC_15_) of andrographolide in an AGS-EBV cell line [15]. In this study, we further determined the ability of andrographolide to induce cytotoxicity in gastric cancer cell lines, AGS and AGS-EBV. Cells were either treated with NaB or andrographolide or the combination of both, and cytotoxicity was determined using CCK-8 assay. Viability of AGS cells was lower when treated with andrographolide and the combination of andrographolide and NaB than with NaB treatment only, and in a time-dependent manner (Figure 6A). Similarly, viability of AGS-EBV cells was also significantly lower in cells treated with andrographolide and the combination of andrographolide and NaB than in those subjected to NaB treatment only, and also in a time-dependent manner (Figure 6B). In addition, we further compared the viability of AGS and AGS-EBV cell lines treated with NaB, andrographolide, or a combination of both. After 48 h of treatment, viability of AGS-EBV cells treated with andrographolide and the combination of andrographolide and NaB was significantly lower than that of AGS cells (Figure 6C). This result suggests that andrographolide has more ability to induce cytotoxicity of the EBVaGC cell line than the EBV-negative gastric cancer cell line.

### 3.5. Andrographolide Induces Apoptosis of the EBVaGC Cell Line

**Figure 7:**
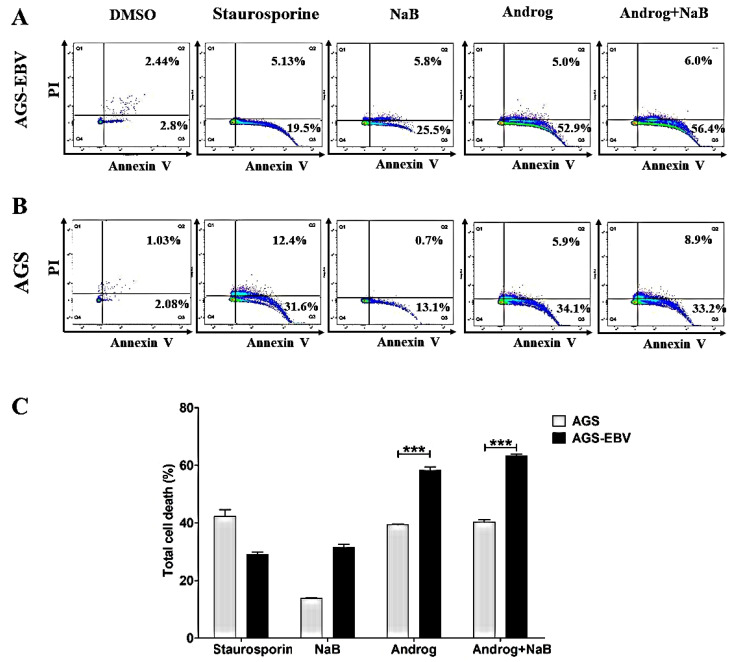
Andrographolide induces apoptosis of GC cells. AGS-EBV and AGS cell lines were treated either with DMSO, NaB, andrographolide, or a combination of andrographolide and NaB. The apoptotic cells were analyzed by flow cytometry. (**A**) Apoptosis in AGS-EBV cells; (**B**) apoptosis in AGS cells; (**C**) comparison of percentages of apoptotic cells in AGS-EBV and AGS cell lines. Staurosporine was used as a positive control for cell apoptosis. ***: *p* < 0.001.

AGS-EBV cells were treated either with DMSO, NaB, andrographolide, or a combination of andrographolide and NaB. LC–MS/MS analysis showed an increase of apoptosis-associated proteins in AGS-EBV cells treated with the combination of andrographolide and NaB. Therefore, we hypothesized that andrographolide can also induce cell death. To test this, we examined apoptosis using flow cytometry. The results showed that andrographolide significantly induced apoptosis of both AGS and AGS-EBV cell lines when compared with DMSO controls and NaB treatment alone (Figure 7A,B). Interestingly, andrographolide induced cell death in AGS-EBV cells at a significantly higher level than in AGS cells (Figure 7C). In addition, our proteomic analysis revealed that the apoptosis-associated proteins were significantly increased in AGS-EBV cells treated with andrographolide relative to other treatments (Table 3). In addition, the expression of pro-apoptotic proteins involves the mitochondria-dependent pathway, including BCL2L1, ENDOG, and PUMA. Therefore, this result demonstrates that andrographolide induces apoptosis of gastric cancer cell lines, especially EBV-positive gastric cancer cells, probably via the mitochondria-dependent pathway.

**Figure 8:**
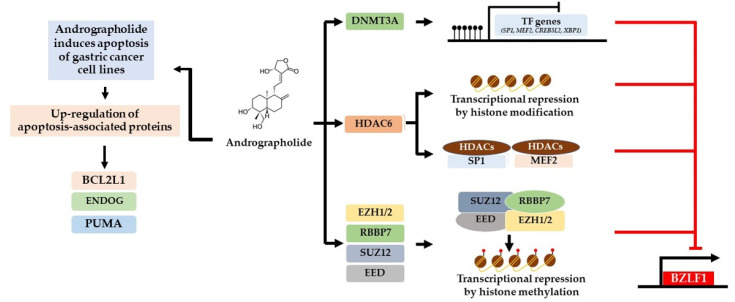
Possible mechanisms of inhibition of EBV lytic reactivation and induction of apoptosis by andrographolide. For the inhibition of lytic reactivation of EBV, there are three possible mechanisms. The first is by inducing the expression of DNA methyltransferases (DNMT3A) that may inhibit the expression of activator proteins (MEF2D, SP1, and others) through DNA methylation that may lead to the suppression of BZLF1. Secondly, andrographolide induces the expression of histone deacetylases, HDAC6. HDAC6 may inhibit the expression of host TFs through histone acetylation or interact directly with host TFs (MEF2D and SP1), interfering with their ability to activate the EBV lytic reactivation. Lastly, andrographolide induces the expression of PRC2-complex proteins (EZH1/2, RBBP7, SUZ12, and EED). These proteins may disrupt the transcriptional activity of EBV. In addition, andrographolide treatment can induce the expression of apoptotic-related proteins, especially protein involved in mitochondria dependent pathways (BCL2L1, EDOG, and PUMA).

Collectively, our study demonstrated that andrographolide inhibits EBV lytic reactivation in EBVaGC cells by inhibition of the expression of EBV lytic proteins and of certain host TFs. In contrast, andrographolide can induce the epigenetic mechanism that may regulate the expression of TFs (Figure 8). In addition, andrographolide can induce death of GC cells and EBVaGC cells via apoptosis.

## 4. Discussion

Andrographolide is active against various viruses, such as herpes simplex virus type 1 (HSV-1) [28], influenza virus [29], human immunodeficiency virus (HIV) [30], CHIKV [10], and DENV [9]. In addition, andrographolide can inhibit EBV lytic genes through the inhibition of Zta promoter activity. Our previous study also demonstrated that andrographolide inhibited EBV lytic reactivation by the inhibition of lytic protein expression and of EBV virion production. However, the molecular mechanism by which andrographolide inhibits EBV reactivation has been unclear.

As mentioned earlier, EBV has two infection phases: lytic and latent. EBV lytic reactivation is mediated by host TFs that can repress or stimulate transcription of Zp and Rp. The Zp promoter contains the cis-acting motifs required for Zp activation. Host TFs, including MEF2D, SP1, SP3, CREB, ATF-1, the ATF-2/c-jun heterodimer, and AP1, can bind specifically to their responsive elements and activate Zp [31,32,33,34]. On the other hand, the activity of Zp is restricted by repressive factors, including Jun dimerization protein 2 (JDP2) [35], zinc finger E-box binding factor (ZEB) [36], Yin Yang 1 (YY-1) [37], and sumoylation of *BZLF1* [38].

Consistently with this, we observed down-regulation of several host TFs, including CREB3L2, MEF2D, SP1, XBP1, and XPB, in AGS-EBV cells treated with andrographolide and in which lytic reactivation was subsequently induced by NaB compared with NaB treatment alone. In contrast, the expression of epigenetic mechanisms, including DNA methylation (via DNMT3A), histone acetylation (via HDAC6 and HDAC9), and histone methylation (EED, EZH1, EZH2, RBBP7, SUZ12, and HIRA), were significantly increased in AGS-EBV cells treated with the combination of andrographolide and NaB when compared with NaB treatment alone. Therefore, the inhibition of EBV lytic reactivation may be mediated by epigenetic mechanisms.

Accumulating evidence suggests that histone modification plays an important role in the inhibition of EBV lytic reactivation. The alteration of histone acetylation at the promoter for the BZLF1 gene determines the potential for EBV lytic reactivation [39]. Treatment with histone deacetylase inhibitors (HDACi), TPA, NaB, trichostatin A (TSA), and valproic acid (VPA), could induce EBV lytic reactivation in Raji, B95-8, and HH514-16 cells by disruption of HDACs [40]. The levels of histone trimethylases, including histone H3 lysine 27 trimethylation (H3K27me3), H3K9me2/3, and H4K20me3, and their effects on the Zp were increased in EBV latently infected Raji cells. In addition, high levels of histone acetylation and of H3K4me3 markers were well correlated with EBV lytic reactivation [41]. EZH2, a major histone H3 lysine 27 (K27) methyltransferase, plays a critical role in the inhibition of lytic replication of EBV in Akata-EBV cells [42]. In addition, histone chaperones, CAF1 and HIRA, play key roles in maintenance of EBV latency by introduction of H3K9me3 and H3K27me3 to the lytic cycle regulatory elements in Akata-EBV and P3HR1 cells [43]. Consistently with this, we also found that the expression of PRC2 complex proteins, including EED, EZH1, EZH2, RBBP7, and SUZ12, and the histone chaperone HIRA, was significant up-regulated in AGS-EBV cells treated with andrographolide and with subsequent induction of lytic reactivation by NaB. Therefore, epigenetic mechanisms, especially histone modifications, play crucial roles in the inhibition of EBV lytic reactivation in EBVaGC cells.

In addition to histone modification, HDACs may directly bind with host TFs. In this study, by using a protein–protein interaction prediction approach (PSOPIA), we found that HDAC6 and HDAC9 can directly interact with MEF2D and SP1. This agrees with studies showing that other HDACs can directly interact with MEF2D or SP1. SP1 and SP3 directly bind to specific consensus GC-rich sequences in the HDAC4 promoter to drive HDAC4 transcription [44]. Sp1 is a target for HDAC1-mediated transcriptional repression. The interaction between SP1 and HDAC1 is direct and requires the carboxy-terminal domain of SP1 [45]. In addition, HDAC3, HDAC4, HDAC5, HDAC7, and HDAC9 are known to recognize primarily the MEF2-specific domain, but HDAC3 interacts directly with the N-terminal region contains the DNA-binding domain (MADS box) of MEF2D. In addition, HDAC3 is associated with the acetyltransferases p300 and p300/CBP-associated factor (PCAF) to reverse autoacetylation [46].

In the present study, we assessed the ability of andrographolide to induce cytotoxicity in the gastric cancer cell lines AGS and AGS-EBV. As expected, andrographolide combined with NaB induced cytotoxicity in both AGS and AGS-EBV cells when compared with NaB treatment alone. In addition, viability of AGS-EBV cells that were treated with andrographolide was significantly lower than for AGS cells. Consistent with this, andrographolide significantly induced apoptosis in gastric cancer cell lines, especially AGS-EBV. Previous studies have demonstrated that andrographolide can induce cell-cycle arrest and apoptosis of cancer cells by inhibiting cellular pathways such as phosphoinositide 3-kinase/protein kinase B, mitogen-activated protein kinase, and other tumor growth pathways, depending on the type of treated cells [47,48,49]. In addition, andrographolide can trigger intrinsic and extrinsic apoptotic pathways in various cancer cells via the activation of p53, reactive oxygen species (ROS), and topoisomerase II [50,51,52]. The induction of apoptosis by andrographolide is mediated through a mitochondria-dependent pathway by induction of pro-apoptotic proteins, including Bax, HL-60, and mitochondrial cytochrome c [53]. In addition, andrographolide can also induce ROS-dependent apoptosis in lymphoma cell lines and in primary tumor samples, and the mechanism appears to proceed through intrinsic and extrinsic caspase pathways and is associated with Bax/Bak mitochondrial translocation [54]. Therefore, the induction of apoptosis by andrographolide may be mediated through the mitochondria-dependent pathway.

In this present study, we investigated the effects of andrographolide on the inhibition of lytic reactivation of EBV and induced apoptosis in gastric cancer cells (in vitro), which cannot be clearly clinically linked. In previous reports, EBV lytic reactivation could induce cellular genomic instability by induction of micronuclei and γH2AX expression, markers of DNA damage and genomic instability, and enhancement of the tumorigenicity of NPC [55,56] and was highly correlated with cancer progression, poor prognosis, and tumor recurrence in various human malignancies [57]. Therefore, inhibition of lytic reactivation of EBV might be beneficial for prognosis and outcomes.

## 5. Conclusions

This study demonstrates for the first time that andrographolide can inhibit EBV lytic reactivation by modulating the expression of viral and cellular proteins, especially TFs (SP1 and MEF2). The expression of TFs can probably be regulated via processes including DNA methylation, histone acetylation, and histone methylation. Interestingly, HDAC6 can directly interact with TFs, which inhibit the transcriptional activity of these TFs. In addition, andrographolide also induces cytotoxicity and apoptosis in GC and EBVaGC cells.

## Figures and Tables

**Table 1:** List of primers used in this study.

Genes	Primer Sequences (5′–3′)	
*MEF2D*	F	CATGCCCACTGCCTACAACA
	R	TGACATTGCCTAGCGACAGC
*SP1*	F	GACAGTGAAGGAAGGGGCTC
	R	AATGGCCTCTCGCCTGTATG
*HDAC5*	F	CCTCAACCATTCCCTCCCAC
	R	GTTCAGAGGCTGTTTTGCGG
*HDAC6*	F	TGGCTATTGCATGTTCAACCA
	R	GTCGAAGGTGAACTGTGTTCCT
*HDAC9*	F	CCCCTGCTGCCTCTGTTTTA
	R	GGAATTGCCACAAACGCACT
*GAPDH*	F	TCATCAGCAATGCCTCCTGCA
	R	TGGGTGGCAGTGATGGCA

**Table 2:** The estimated probabilities that HDAC6 and HDAC9 can directly interact with MEF2D and SP1, as determined using a PPI prediction tool.

Interaction of Proteins	The Probability of Protein–Protein Interaction			
	S_seg_	S_dom_	S_net_	S_all_
HDAC6–MEF2D	0.9884	0.5146	0.8351	0.9922
HDAC6–SP1	0.6846	0.3897	0.8351	0.3417
HDAC9–MEF2D	0.9962	0.9965	0.8351	0.9991
HDAC9–SP1	0.8130	0.5146	0.8351	0.9011

**Table 3:** List of apoptosis-associated proteins up-regulated in andrographolide-treated cells.

Protein ID	Protein Name	Protein Symbol	Fold Increase
O75962	Triple functional domain protein	TRIO	1.89
P32248	Chemokine receptor type 7	CCR7	2.76
P62873	Guanine nucleotide-binding protein G(I)/G(S)/G(T) subunit beta-1	GNB1	3.39
F4I6D6	RNA polymerase I-specific transcription initiation factor RRN3	RRN3	13.59
Q16594	Transcription initiation factor TFIID subunit 9	TAF9	13.09
O00755	Protein Wnt-7a	WNT7A	15.96
Q13489	Baculoviral IAP repeat-containing protein 3	BIRC3	12.79
O14757	Serine/threonine-protein kinase Chk1	CHEK1	12.35
O00115	Deoxyribonuclease-2-alpha	DNASE2	3.29
Q9NPG1	Frizzled class receptor 3	FZD3	14.08
O43424	Glutamate ionotropic receptor delta type subunit 2	GRID2	5.13
O00198	Activator of apoptosis harakiri	HRK	13.74
P05981	Serine protease hepsin	HPN	12.36
Q53GA4	Pleckstrin homology-like domain family A member 2	PHLDA2	2.11
P07237	Protein disulfide-isomerase	P4HB	14.79
Q13591	Semaphorin-5A	SEMA5A	13.11
Q07817	Bcl-2-like protein 1	BCL2L1	2.42
O43521	Bcl-2-like protein 11	BCL2L11	11.48
Q13164	Mitogen-activated protein kinase 7	MAPK7	3.07
O95819	Mitogen-activated protein 4 kinase 4	MAP4K4	2.04
Q06643	Lymphotoxin-beta	LTB	6.77
P08648	Integrin subunit alpha-5	ITGA5	5.47
Q14974	Importin subunit beta-1	KPNB1	4.81
Q9GZU2	Paternally-expressed gene 3 protein	PEG3	3.60
Q14432	cGMP-inhibited 3′,5′-cyclic phosphodiesterase A	PDE3A	14.85
Q14249	Endonuclease G	ENDOG	13.32
Q96PG8	p53 up-regulated modulator of apoptosis	PUMA	15.36

## Data Availability

The published article includes all datasets generated or analyzed during this study.

## Supplementary Materials

The following are available online at https://www.mdpi.com/article/10.3390/microorganisms9122561/s1, Table S1: The positions of HDAC6 interacts either with MEF2D or SP1.

## References

[1] Fukayama M., Hino R., Uozaki H. (2008). Epstein-Barr virus and gastric carcinoma: Virus-host interactions leading to carcinoma. Cancer Sci..

[2] Kenney S.C., Mertz J.E. (2014). Regulation of the latent-lytic switch in Epstein-Barr virus. Sem. Cancer Biol..

[3] Murata T. (2014). Regulation of Epstein-Barr virus reactivation from latency. Microbiol. Immunol..

[4] Chiu S.H., Wu C.C., Fang C.Y., Yu S.L., Hsu H.Y., Chow Y.H., Chen J.Y. (2014). Epstein-Barr virus BALF3 mediates genomic instability and progressive malignancy in nasopharyngeal carcinoma. Oncotarget.

[5] Okhuarobo A., Ehizogie Falodun J., Erharuyi O., Imieje V., Falodun A., Langer P. (2014). Harnessing the medicinal properties of Andrographis paniculata for diseases and beyond: A review of its phytochemistry and pharmacology. Asian Pac. J. Trop. Dis..

[6] Sareer O., Ahad A., Umar S. (2012). Prophylactic and lenitive effects of Andrographis paniculate against common human ailments: An exhaustive and comprehensive reappraisal. J. Pharm. Res..

[7] Akbar S. (2011). Andrographis paniculata: A review of pharmacological activities and clinical effects. Altern. Med. Rev..

[8] Seubsasana S., Pientong C., Ekalaksananan T., Thongchai S., Aromdee C. (2011). A potential andrographolide analogue against the replication of herpes simplex virus type 1 in vero cells. Med. Chem..

[9] Panraksa P., Ramphan S., Khongwichit S., Smith D.R. (2017). Activity of andrographolide against dengue virus. Antivir. Res..

[10] Wintachai P., Kaur P., Lee R.C., Ramphan S., Kuadkitkan A., Wikan N., Smith D.R. (2015). Activity of andrographolide against chikungunya virus infection. Sci. Rep..

[11] Fangkham S., Ekalaksananan T., Aromdee C., Seubsasana S., Kongyingyoes B., Patarapadungkit N., Pientong C. (2012). The effect of andrographolide on Human papillomavirus type 16 (HPV16) positive cervical cancer cells (SiHa). Int. J. Infect. Dis..

[12] Chen H., Ma Y.B., Huang X.Y., Geng C.A., Zhao Y., Wang L.J., Chen J.J. (2014). Synthesis, structure–activity relationships and biological evaluation of dehydroandrographolide and andrographolide derivatives as novel anti-hepatitis B virus agents. Med. Chem. Lett..

[13] Ding Y., Chen L., Wu W., Yang J., Yang Z., Liu S. (2017). Andrographolide inhibits influenza A virus-induced inflammation in a murine model through NF-κB and JAK-STAT signaling pathway. Microbes Infect..

[14] Lin T.P., Chen S.Y., Duh P.D., Chang L.K., Liu Y.N. (2008). Inhibition of the Epstein-Barr virus lytic cycle by andrographolide. Biol. Pharm. Bull..

[15] Aromdee C., Suebsasana S., Ekalaksananan T., Pientong C., Thongchai S. (2011). Stage of action of naturally occurring andrographolides and their semisynthetic analogues against herpes simplex virus type 1 in vitro. Planta Med..

[16] Wu B., Chen X., Zhou Y., Hu P., Wu D., Zheng G., Cai Y. (2018). Andrographolide inhibits proliferation and induces apoptosis of nasopharyngeal carcinoma cell line C666-1 through LKB1-AMPK-dependent signaling pathways. Die Pharm..

[17] Lowry O.H., Rosebrough N.J., Farr A.L., Randall R.J. (1951). Protein measurement with the Folin phenol reagent. J. Biol. Chem..

[18] Tyanova S., Temu T., Sinitcyn P., Carlson A., Hein M.Y., Geiger T., Cox J. (2016). The Perseus computational platform for comprehensive analysis of (prote)omics data. Nat. Methods.

[19] Mi H., Muruganujan A., Huang X., Ebert D., Mills C., Guo X., Thomas P.D. (2019). Protocol Update for large-scale genome and gene function analysis with the PANTHER classification system (v.14.0). Nat. Protoc..

[20] Bardou P., Mariette J., Escudié F., Djemiel C., Klopp C. (2014). Jvenn: An interactive Venn diagram viewer. BMC Bioinform..

[21] Szklarczyk D., Morris J.H., Cook H., Kuhn M., Wyder S., Simonovic M., von Mering C. (2017). The STRING database in 2017: Quality-controlled protein-protein association networks, made broadly accessible. Nucleic Acids Res. Spec. Publ..

[22] Waterhouse A., Bertoni M., Bienert S., Studer G., Tauriello G., Gumienny R., Schwede T. (2018). SWISS-MODEL: Homology modelling of protein structures and complexes. Nucleic Acids Res..

[23] Comeau S.R., Gatchell D.W., Vajda S., Camacho C.J. (2004). ClusPro: An automated docking and discrimination method for the prediction of protein complexes. Bioinformatics.

[24] Kozakov D., Hall D.R., Xia B., Porter K.A., Padhorny D., Yueh C., Vajda S. (2017). The ClusPro web server for protein-protein docking. Nat. Protoc..

[25] Chen H.P., Zhao Y.T., Zhao T.C. (2015). Histone deacetylases and mechanisms of regulation of gene expression. Crit. Rev. Oncog..

[26] Li G., Tian Y., Zhu W.-G. (2020). The roles of histone deacetylases and their inhibitors in cancer therapy. Cell Dev..

[27] Grunstein M. (1997). Histone acetylation in chromatin structure and transcription. Nature.

[28] Wiart C., Kumar K., Yusof M.Y., Hamimah H., Fauzi Z.M., Sulaiman M. (2005). Antiviral properties of ent-labdene diterpenes of Andrographis paniculata nees, inhibitors of herpes simplex virus type 1. Phytother. Res..

[29] Chen J.X., Xue H.J., Ye W.C., Fang B.H., Liu Y.H., Yuan S.H., Wang Y.Q. (2009). Activity of andrographolide and its derivatives against influenza virus in vivo and in vitro. Biol. Pharm. Bull..

[30] Uttekar M.M., Das T., Pawar R.S., Bhandari B., Menon V., Nutan Bhat S.V. (2012). Anti-HIV activity of semisynthetic derivatives of andrographolide and computational study of HIV-1 gp120 protein binding. Eur. J. Med. Chem..

[31] Lu J., Chen S.Y., Chua H.H., Liu Y.S., Huang Y.T., Chang Y., Tsai C.H. (2000). Upregulation of tyrosine kinase TKT by the Epstein-Barr virus transactivator Zta. J. Virol..

[32] Liu S., Liu P., Borras A., Chatila T., Speck S.H. (1997). Cyclosporin A-sensitive induction of the Epstein-Barr virus lytic switch is mediated via a novel pathway involving a MEF2 family member. EMBO J..

[33] Yu X., Wang Z., Mertz J.E. (2007). ZEB1 regulates the latent-lytic switch in infection by Epstein-Barr virus. PLoS Pathog..

[34] Adamson A.L., Darr D., Holley-Guthrie E., Johnson R.A., Mauser A., Swenson J., Kenney S. (2000). Epstein-Barr virus immediate-early proteins BZLF1 and BRLF1 activate the ATF2 transcription factor by increasing the levels of phosphorylated p38 and c-Jun N-terminal kinases. J. Virol..

[35] Flemington E., Speck S.H. (1990). Autoregulation of Epstein-Barr virus putative lytic switch gene BZLF1. J. Virol..

[36] Murata T., Noda C., Saito S., Kawashima D., Sugimoto A., Isomura H., Tsurumi T. (2011). Involvement of Jun dimerization protein 2 (JDP2) in the maintenance of Epstein-Barr virus latency. J. Biol. Chem..

[37] Montalvo E.A., Cottam M., Hill S., Wang Y.J. (1995). YY1 binds to and regulates cis-acting negative elements in the Epstein-Barr virus BZLF1 promoter. Virol. J..

[38] Murata T., Hotta N., Toyama S., Nakayama S., Chiba S., Isomura H., Tsurumi T. (2010). Transcriptional repression by sumoylation of Epstein-Barr virus BZLF1 protein correlates with association of histone deacetylase. J. Biol. Chem..

[39] Jenkins P.J., Binné U.K., Farrell P.J. (2000). Histone acetylation and reactivation of Epstein-Barr virus from latency. Virol. J..

[40] Countryman J.K., Gradoville L., Miller G. (2008). Histone hyperacetylation occurs on promoters of lytic cycle regulatory genes in Epstein-Barr virus-infected cell lines which are refractory to disruption of latency by histone deacetylase inhibitors. J. Virol..

[41] Murata T., Kondo Y., Sugimoto A., Kawashima D., Saito S., Isomura H., Tsurumi T. (2012). Epigenetic Histone Modification of Epstein-Barr Virus BZLF1 Promoter during Latency and Reactivation in Raji Cells. Virol. J..

[42] Ichikawa T., Okuno Y., Sato Y., Goshima F., Yoshiyama H., Kanda T., Murata T. (2018). Regulation of Epstein-Barr virus life cycle and cell proliferation by histone H3K27 methyltransferase EZH2 in Akata Cells. mSphere.

[43] Zhang Y., Jiang C., Trudeau Stephen J., Narita Y., Zhao B., Teng M., Damania B. (2020). Histone loaders CAF1 and HIRA restrict Epstein-Barr virus B-cell lytic reactivation. mBio.

[44] Liu F., Pore N., Kim M., Voong K.R., Dowling M., Maity A., Kao G.D. (2006). Regulation of histone deacetylase 4 expression by the SP family of transcription factors. Mol. Biol. Cell.

[45] Doetzlhofer A., Rotheneder H., Lagger G., Koranda M., Kurtev V., Brosch G., Seiser C. (1999). Histone deacetylase 1 can repress transcription by binding to Sp1. Mol. Cell. Biol..

[46] Grégoire S., Xiao L., Nie J., Zhang X., Xu M., Li J., Yang X.J. (2007). Histone deacetylase 3 interacts with and deacetylates myocyte enhancer factor 2. Mol. Cell. Biol..

[47] Li J., Zhang C., Jiang H., Cheng J. (2015). Andrographolide inhibits hypoxia-inducible factor-1 through phosphatidylinositol 3-kinase/AKT pathway and suppresses breast cancer growth. OncoTargets Ther..

[48] Liu S.H., Lin C.H., Liang F.P., Chen P.F., Kuo C.D., Alam M.M., Fu S.L. (2014). Andrographolide downregulates the v-Src and Bcr-Abl oncoproteins and induces Hsp90 cleavage in the ROS-dependent suppression of cancer malignancy. Biochem. Pharmacol..

[49] Shen K., Ji L., Lu B., Xu C., Gong C., Morahan G., Wang Z. (2014). Andrographolide inhibits tumor angiogenesis via blocking VEGFA/VEGFR2-MAPKs signaling cascade. Chem. Biol. Interact..

[50] Lu C.Y., Yang Y.C., Li C.C., Liu K.L., Lii C.K., Chen H.W. (2014). Andrographolide inhibits TNFα-induced ICAM-1 expression via suppression of NADPH oxidase activation and induction of HO-1 and GCLM expression through the PI3K/Akt/Nrf2 and PI3K/Akt/AP-1 pathways in human endothelial cells. Biochem. Pharmacol..

[51] Nateewattana J., Dutta S., Reabroi S., Saeeng R., Kasemsook S., Chairoungdua A., Piyachaturawat P. (2014). Induction of apoptosis in cholangiocarcinoma by an andrographolide analogue is mediated through topoisomerase II alpha inhibition. Eur. J. Pharmacol..

[52] Yang S.H., Wang S.M., Syu J.P., Chen Y., Wang S.D., Peng Y.S., Kung H.N. (2014). Andrographolide induces apoptosis of C6 glioma cells via the ERK-p53-caspase 7-PARP pathway. Biomed. Res. Int..

[53] Cheung H.Y., Cheung S.H., Li J., Cheung C.S., Lai W.P., Fong W.F., Leung F.M. (2005). Andrographolide Isolated from Andrographis paniculata Induces cell cycle arrest and mitochondrial-mediated apoptosis in human leukemic HL-60 cells. Planta Med..

[54] Wu C.C., Fang C.Y., Cheng Y.J., Hsu H.Y., Chou S.P., Huang S.Y., Chen J.Y. (2018). Inhibition of Epstein-Barr virus reactivation by the flavonoid apigenin. J. Biomed. Sci..

[55] Wu C.C., Fang C.Y., Huang S.Y., Chiu S.H., Lee C.H., Chen J.Y. (2018). Perspective: Contribution of Epstein-Barr virus (EBV) Reactivation to the Carcinogenicity of Nasopharyngeal Cancer Cells. Cancers.

[56] Yang S., Evens A.M., Prachand S., Singh A.T.K., Bhalla S., David K., Gordon L.I. (2010). Mitochondrial-mediated apoptosis in lymphoma cells by the diterpenoid lactone andrographolide, the active component of Andrographis paniculata. Clin. Cancer Res..

[57] Fang C.Y., Lee C.H., Wu C.C., Chang Y.T., Yu S.L., Chou S.P., Chen J.Y. (2009). Recurrent chemical reactivations of EBV promotes genome instability and enhances tumor progression of nasopharyngeal carcinoma cells. Int. J. Cancer.

