# Supplementary material for: Andrographolide Inhibits Lytic Reactivation of Epstein-Barr Virus by Modulating Transcription Factors in Gastric Cancer
Source: Microorganisms. 2021 Dec 10;9(12):2561. doi: 10.3390/microorganisms9122561 (PMC8708910; doi:10.3390/microorganisms9122561)
Supplement: Supplementary file 1 [file microorganisms-09-02561-s001.zip › microorganisms-1490382-supplementary.pdf]

Table S1. The positions of HDAC6 interacts with either MEF2D or SP1 via hydrogen bond.

No.	HDAC6:MEF2D	HDAC6:SP1
1	ARG24:HH11-GLU34:OE1	ARG654:HH22-ASP675:OD2
2	ARG24:HH21-GLU34:OE1	ARG654:HH22-ASP675:OD2
3	LYS25:HZ1-GLU77:OE1	ARG654:HH22-ASP675:OD2
4	LYS25:HZ3-GLU77:OE2	ARG654:HH22-ASP675:OD2
5	LYS53:HZ1-GLU74:OE1	ARG654:HH22-ASP675:OD2
6	LYS53:HZ3-GLU74:OE2	ARG654:HH22-ASP675:OD2
7	GLU452:OE1-ARG3:HH21	ARG654:HH22-ASP675:OD2
8	GLU459:OE2-LYS23:HZ1	ARG654:HH22-ASP675:OD2
9	GLU34:OE1-ARG24:HH11	ARG654:HH22-ASP675:OD2
10	GLU34:OE1-ARG24:HH21	ARG654:HH22-ASP675:OD2
11	GLU77:OE1-LYS25:HZ1	ARG654:HH22-ASP675:OD2
12	GLU77:OE2-LYS25:HZ3	ARG654:HH22-ASP675:OD2
13	GLU461:OE2-LYS30:HZ1	ARG654:HH22-ASP675:OD2
14	GLU74:OE1-LYS53:HZ1	ARG654:HH22-ASP675:OD2
15	GLU74:OE2-LYS53:HZ3	ARG654:HH22-ASP675:OD2
16	ILE11:HN-LEU38:O	ARG654:HH22-ASP675:OD2
17	SER36:HG-ASN81:OD1	ARG654:HH22-ASP675:OD2
18	GLU42:HN-PHE48:O	ARG654:HH22-ASP675:OD2
19	ALA44:HN-ILE46:O	ARG654:HH22-ASP675:OD2
20	ILE46:HN-ALA44:O	ARG654:HH22-ASP675:OD2
21	PHE48:HN-GLU42:O	ARG654:HH22-ASP675:OD2
22	HIS50:HN-ASP40:O	ARG654:HH22-ASP675:OD2
23	HIS50:HD1-CYS39:O	ARG654:HH22-ASP675:OD2
24	HIS50:HD1-ASP40:O	ARG654:HH22-ASP675:OD2
25	LYS53:HZ1-PRO75:O	ARG654:HH22-ASP675:OD2
26	LYS53:HZ2-TYR72:OH	ARG654:HH22-ASP675:OD2
27	GLN56:HN-GLU77:O	ARG654:HH22-ASP675:OD2
28	GLN56:HE21-SER78:OG	ARG654:HH22-ASP675:OD2
29	GLN56:HE22-TYR69:OH	ARG654:HH22-ASP675:OD2
30	ALA58:HN-ARG79:O	ARG654:HH22-ASP675:OD2
31	LYS68:HZ3-ASN52:OD1	ARG654:HH22-ASP675:OD2
32	TYR69:HH-ASP63:OD1	ARG654:HH22-ASP675:OD2
33	GLU77:HN-LEU54:O	ARG654:HH22-ASP675:OD2
34	ARG79:HN-GLN56:O	ARG654:HH22-ASP675:OD2
35	THR80:HG1-THR60:O	ARG654:HH22-ASP675:OD2
36	ASN81:HN-SER59:O	ARG654:HH22-ASP675:OD2
37	ASP40:OD1-ARG10:HE	ARG654:HH22-ASP675:OD2
38	LEU38:O-ILE11:HN	ARG654:HH22-ASP675:OD2
39	CYS39:O-ARG17:HE	ARG654:HH22-ASP675:OD2
40	GLU457:O-LYS23:HZ3	ARG654:HH22-ASP675:OD2
41	PHE48:O-GLU42:HN	ARG654:HH22-ASP675:OD2
42	ILE46:O-ALA44:HN	ARG654:HH22-ASP675:OD2

43	ALA44:O-ILE46:HN	ARG654:HH22-ASP675:OD2
44	GLU42:O-PHE48:HN	ARG654:HH22-ASP675:OD2
45	ASP40:O-HIS50:HN	ARG654:HH22-ASP675:OD2
46	ASP40:OD2-HIS50:HD1	ARG654:HH22-ASP675:OD2
47	PRO75:O-LYS53:HZ1	
48	TYR72:OH-LYS53:HZ2	
49	GLU77:O-GLN56:HN	
50	SER78:OG-GLN56:HE21	
51	TYR69:OH-GLN56:HE22	
52	ARG79:O-ALA58:HN	
53	ASN52:OD1-LYS68:HZ3	
54	ASP63:OD1-TYR69:HH	
55	LEU54:O-GLU77:HN	
56	GLN56:O-ARG79:HN	
57	THR60:O-THR80:HG1	
58	ALA58:O-ASN81:HN	
59	SER59:O-ASN81:HN	
60	SER59:O-ASN81:HD21	
61	ARG10:HA-VAL37:O	
62	ARG10:HA-LEU38:O	
63	ARG17:HD1-LEU38:O	
64	TYR33:HA-ASN81:OD1	
65	LEU38:HA-GLN9:O	
66	CYS41:HA-PHE48:O	
67	ILE43:HA-ILE46:O	
68	LEU45:HA-ALA44:O	
69	HIS50:HE1-CYS39:O	
70	LYS53:HE2-PRO75:O	
71	PHE55:HA-GLU77:OE2	
72	PHE55:HA-GLU77:O	
73	TYR57:HA-ARG79:O	
74	LYS68:HE1-ASN52:OD1	
75	SER78:HA-GLN56:O	
76	THR80:HA-ALA58:O	
77	VAL37:O-ARG10:HA	
78	LEU38:O-ARG10:HA	
79	LEU38:O-ARG17:HD1	
80	GLU459:OE2-LYS23:HE1	
81	GLU465:OE1-LYS30:HE2	
82	ASN81:OD1-TYR33:HA	
83	GLN9:O-LEU38:HA	
84	PHE48:O-CYS41:HA	
85	ILE46:O-ILE43:HA	
86	ALA44:O-LEU45:HA	
87	GLU42:O-ILE47:HA	
88	PRO75:O-LYS53:HE2	

89	GLU77:OE2-PHE55:HA
90	GLU77:O-PHE55:HA
91	ASN52:OD1-LYS68:HE1
92	GLN56:O-SER78:HA
93	ALA58:O-THR80:HA
